# Supplementary material for: ECA-VFog: An efficient certificateless authentication scheme for 5G-assisted vehicular fog computing
Source: PLoS One. 2023 Jun 23;18(6):e0287291. doi: 10.1371/journal.pone.0287291 (PMC10289398; doi:10.1371/journal.pone.0287291)
Supplement: Attachment — Submitted filename: Response to Reviewers.docx [file pone.0287291.s001.docx]

Original Manuscript ID: PONE-D-23-13861

Original Article Title: “ECA-VFog: An Efficient Certificateless Authentication Scheme for 5G-Assisted Vehicular Fog Computing”

To: PLOS ONE

Re: Response to reviewers

Dear Editor,

Thank you for allowing a resubmission of our manuscript, with an opportunity to address the reviewers’ comments.

Best regards,

Dr. Mahmood A. Al-Shareeeda.

Reviewer 1: Concern #1: Section number must be given for all the sections and sub-sections.

Reviewer 1: Response #1: Thanks for the valuable comments. We revise the manuscript by adding sections and sub-sections as follows.

….

Reviewer 1: Concern #2: Why have the authors focused on 5G? Because nowadays many researcher have started on 6G.

Reviewer 1: Response #2: Thanks for the valuable comments. The 5G is the scope of this research. In future work, we extend this work to apply 6G instead of 5G for secure compunctions. The revised sentence has been added in conclusion section of the paper as follows.

Reviewer 1: Concern #3: There are few typographical mistakes. For example, in the Introduction section, “this study is” must be “This study is”. In the same section, “Section reviews some related work” must be “Section 2 reviews some related work”. Modify the entire paragraph.

Reviewer 1: Response #3: Thanks for the valuable comments. We revise the manuscript by modifying the entire paragraph as follows.

Reviewer 1: Concern #4:  The authors must give expansion for all the abbreviations. For example check ECA.

Reviewer 1: Response #4: Thanks for the valuable comments. We revise the manuscript by adding missing definition of abbreviations in the following table.

Reviewer 1: Concern #5:  Use unique symbols in the entire paper. For example, P is not italic in one place and it is italic in another place under the subheading Elliptic Curve Cryptography (ECC).

Reviewer 1: Response #5: Thanks for the valuable comments. We revise the manuscript by remodify entire subheading Elliptic Curve Cryptography (ECC) as follows.

Reviewer 1: Concern #6:  Related work section is weak. Because Some important and recent references are missing, the following references must be totally added in the Section "References" (otherwise, the reference is not enough, then it must be revised again until it is enough):

Reviewer 1: Response #6: Thanks for the valuable comments. We revise the manuscript by reviewing the relevant articles as follows.

Xu et al. [27] constructed certificateless designated verifier proxy signature using unmanned aerial vehicles (UAVs) to address privacy and security concerns in smart city systems. Ming et al. [28] designed an efficient certificateless authentication scheme by achieving a security-enhanced solution and addressing massive communication overhead, security vulnerability, and computational complexity. Tan et al. [29] proposed a certificateless group authentication scheme based on UAV in order to achieve security communication in infrastructure-less internet of vehicle (IoV). Zhou et al. [30] designed a secure ECC scheme by using key agreement and a three-party authentication scheme in medical IoT. Rajasekaran et al. [31] proposed a secure ECC method that supports batch verification and mutual authentication for online learning in Industry 4.0.

-------------------------------------------------------------------------------------------------------------------------------------

Reviewer 2: Concern #1:  There are many errors in the abbreviation format of words in the article, for example, the full name of VANET is not explained for the first time. In addition, the full names of PKI and ID also appear several times. Therefore, it is necessary to correct the abbreviation errors that appear in the paper as a whole.

Reviewer 2: Response #1: Thanks for the valuable comments. We revise the manuscript by defining the abbreviation in Table and introduction section as it first appears.

Reviewer 2: Concern #2:  The literature annotation in this paper is quite arbitrary. For example, references [1-11] are not very relevant to the tagged sentences. However, in the third and fourth paragraphs of the introduction, there is a lack of literature sources when presenting related work. Therefore, it is very necessary to carefully review the appropriateness of each cited paper. In addition, the missing references need to be supplemented.

Reviewer 2: Response #2: Thanks for the valuable comments. In this manuscript, we really focus on updated references that are relevant to our work. We revise the manuscript by adding more references based on suggested reviewers as follows.

Xu et al. [27] constructed certificateless designated verifier proxy signature using unmanned aerial vehicles (UAVs) to address privacy and security concerns in smart city systems. Ming et al. [28] designed an efficient certificateless authentication scheme by achieving a security-enhanced solution and addressing massive communication overhead, security vulnerability, and computational complexity. Tan et al. [29] proposed a certificateless group authentication scheme based on UAV in order to achieve security communication in infrastructure-less internet of vehicle (IoV). Zhou et al. [30] designed a secure ECC scheme by using key agreement and a three-party authentication scheme in medical IoT. Rajasekaran et al. [31] proposed a secure ECC method that supports batch verification and mutual authentication for online learning in Industry 4.0.

Reviewer 2: Concern #3:  In the introduction section, the main contributions of the paper are introduced without elaborating on the innovative ideas of the paper. In particular, in the main contribution section, the differences with existing work are not reflected, and the innovative work of the paper is not described.

Reviewer 2: Response #3: Thanks for the valuable comments. Unlike the related work, this paper is based on the fog computing and the 5G technology to trad-off of between security and performance. Hence, we revise the manuscript by adding the innovative of the proposal as follows.

Reviewer 2: Concern #4:  In the related work, the author lists the current work, but the analysis of its shortcomings is too few. In addition, there are few related works on certificateless authentication schemes that are most relevant to the proposed scheme, and only three papers cannot fully reflect the current research progress.

Reviewer 2: Response #4: Thanks for the valuable comments. We revise the manuscript by adding some relevant certificatesless-based schemes. Seven relevant researches have been reviewed in this subsection as follows.

Reviewer 2: Concern #5:   In the system model section, TRA,KGC,FS and 5G-BS are assumed to be fully trusted, which is obviously completely impossible in the real scenario. Thus, it makes sense to at least assume that the third party is semi-trusted.

Reviewer 2: Response #5: Thanks for the valuable comments. We revise the manuscript by adding the assumption of each unit in the architecture model as follows.

Reviewer 2: Concern #6:  In the section of the ECA-VFog scheme, there is no detailed innovative explanation. Neither based on innovation theory nor using new technologies.

Reviewer 2: Response #6: Thanks for the valuable comments. We revise the manuscript by adding innovation explanation (unlike the related work) as follows.

Reviewer 2: Concern #7: It is suggested to prove the security of the proposed scheme in the standard model or the random oracle model.

Reviewer 2: Response #7: Thanks for the valuable comments. In the related work, the prove of the security based on formal and informal analysis. Therefore, this paper uses the AVISPA simulator tool as standard model to show security attacks resistances.

Reviewer 2: Concern #8:   In the whole article, the integration of 5G technology and Internet of vehicles is not reflected.

Reviewer 2: Response #8: Thanks for the valuable comments. 5G doesn’t compute and save any the security parameters for vehicles. The main aim of 5G is not only to serve a large number of vehicles within its communication range, it also forwards messages among vehicles and fog servers.

-------------------------------------------------------------------------------------------------------------------------------------

Reviewer 3: Concern #1:  In abstract part, the outcome (in numerical) of this proposed have been missing, please add them.

Reviewer 3: Response #1: Thanks for the valuable comments. We revise the manuscript by adding the outcome (in numerical) of this proposed in abstract section.

Reviewer 3: Concern #2:  In related work, the paper strongly required to add some relevant researches such as (An efficient conditional privacy-preserving authentication scheme for the prevention of side-channel attacks in vehicular ad hoc networks)

Reviewer 3: Response #2: Thanks for the valuable comments. We revise the manuscript by reviewing this reference as follows.

Reviewer 3: Concern #3:  What are the benefit from 5G and fog computing? This needs to be clarified in the paper

Reviewer 3: Response #3: Thanks for the valuable comments. We revise the manuscript by adding the benefit from fog computing and 5G in system model as follows.

-5G-Base Station (5G-BS): The 5G-BSs are stationary base stations set up by the side of the road. Its only use is as a bridge between vehicles, fog servers, and TRA, and it lacks both computing and storage capabilities. This is because it can accommodate a wide variety of device-to-device (D2D) communication standards. Because 5G-BSs are hardware, they are immune to attacks.

-Fog Server: Fog server is the roadside infrastructure that enables vehicle-to-infrastructure (V2I) communication and can also realize inter-infrastructure (I2I) communication. FSs can simultaneously relay multiple messages collected from vehicles. FSs are stationed in various areas behind 5G-BS, and passing vehicles are made aware of their location. By sharing information, it can also boost circulation in the area covered by 5G-BS.

Reviewer 3: Concern #4:  page 2, line 50-55 >> double checking, there is missing number/name of sections

Reviewer 3: Response #4: Thanks for the valuable comments. We revise the manuscript by adding the missing number as follows.

Reviewer 3: Concern #5:   Table 2, need to align the text

Reviewer 3: Response #5: Thanks for the valuable comments. We revise the manuscript by aligning the table to text of the paper as follows.

Reviewer 3: Concern #6:  Please add future work in conclusion part

Reviewer 3: Response #6: Thanks for the valuable comments. We revise the manuscript by adding future works as follows.

-------------------------------------------------------------------------------------------------------------------------------------
